# Transplantation as disease modifying therapy in the era of gene therapy medicinal products – health policy considerations

**DOI:** 10.1186/s13023-026-04377-4

**Published:** 2026-08-13

**Authors:** Margreet Wagenmakers, Anna Lehman, Caroline den Hoed, Laura van Dussen, Mirjam Langeveld, Sandra Sirrs

**Affiliations:** 1 Department of Internal Medicine, Erasmus MC, Centre for Lysosomal and Metabolic Disease, University Medical Center Rotterdam, Rotterdam, The Netherlands; 2 Adult Metabolic Diseases Clinic, University of British Columbia, Vancouver, Canada; 3 Department of Gastroenterology and Hepatology, Erasmus MC University Medical Center, Rotterdam, The Netherlands; 4 Department of Endocrinology and Metabolism, Amsterdam UMC, Amsterdam Gastroenterology Endocrinology Metabolism Research Institute, University of Amsterdam, Amsterdam, The Netherlands; 5 Division of Endocrinology, Department of Medicine, University of British Columbia, Vancouver, Canada

**Keywords:** Gene therapy medicinal products, Gene therapy, Liver transplantation, Inherited metabolic diseases, Adults, Glycogen storage disease type 1a, Urea cycle defects, Methylmalonic aciduria, Propionic aciduria, Healthcare policy

## Abstract

**Background:**

New gene therapy medicinal products [GTMP] are being considered as alternatives to liver transplant [LTx] for some patients with inherited metabolic diseases [IMDs] but pose unique challenges for health policy makers.

**Methods:**

Published data on LTx and GTMP in human patients with urea cycle defects [UCD], glycogen storage disease type 1a [GSD1a], methylmalonic aciduria [MMA] and propionic aciduria [PA] were reviewed for efficacy, safety, data quality and health policy considerations.

**Results:**

LTx can reduce [MMA, PA] or eliminate [UCD, GSD1a] metabolic decompensation and improve quality of life. Risk of death peaks in the first year but long-term survival post LTx is similar to medical management. Initial data for GTMP show reduction in metabolic decompensation [MMA, PA, UCD] with more modest impacts in GSD1a. Long-term safety and efficacy data [available for LTx] may not be available at the time of market authorization for GTMP. Age is one health policy challenge as clinical trials for GTMP may target one age group but other age groups may request consideration for treatment. Quality concerns regarding data analysis exist for both modalities. Cost effectiveness of LTx is likely to be significantly more favorable than for GTMP. Access limitations are severe for both treatments, with high opportunity costs [price for GTMP, organ availability for LTx] mandating the need to engage the public as stakeholders in addition to patients, families, manufacturers and clinicians.

**Conclusions:**

LTx remains an effective treatment choice in the era of GTMP given the significant health policy challenges associated with these novel therapies.

## Background and introduction

Transplantation in patients with inherited metabolic diseases [IMDs] can be used as supportive therapy or as disease modifying therapy to alter the disease course or even provide cure. Data have suggested that transplantation as a disease modifying therapy for patients with IMDs is underutilized [1] and this is particularly a problem in adults [2]. There is a new variable to consider in the form of novel gene therapy medicinal products [GTMP] as patients and clinicians may delay transplantation, hoping these GTMP will provide a safer alternative. These diverse therapies may be one-time treatments, such as addition of a target gene or gene editing, or ongoing RNA based therapies which stimulate or silence target gene expression or provide direct expression of an enzyme to function as enzyme replacement therapy.

Clinical trials test GTMP against medical management but, in some conditions, LTx is a more appropriate comparator as both modalities offer potential for significant disease modification or even cure. We discuss the role of liver [LTx] transplantation in the era of rapidly advancing GTMP, focusing on four IMDs for which human data on GTMP are available [glycogen storage disease type 1a [GSD1a; OMIM 232,200], mut^0^ methylmalonic acidemia [MMA; OMIM 251,000], propionic acidemia [PA; OMIM 606,054], and urea cycle defects [UCD]. Even though few GTMPs are currently available outside the clinical trial setting, we feel this discussion is timely, as patients and physicians are already considering upcoming GTMP when making decisions on LTx. Similarly, health policy makers must make decisions on which GTMPs should be reimbursed and for which patients. We review information on efficacy and safety of LTx and GTMP from the health policy perspective and discuss challenges such as limitations in available data, access and opportunity costs related to these therapies. We highlight challenges faced by the adult population but do not focus solely on adults as the challenges facing health policy makers require consideration of all age groups to avoid creating inequity in allocation of resources.

## Liver transplantation – efficacy, risks, and outcomes

### General information on LTx

LTx is a highly invasive procedure with short and long term complications typically performed in patients with end-stage liver disease or liver cancer which impacts outcomes. However, even with these caveats, transplantation outcomes are good, with a 1-year survival of 90% in children and 88% in adults and a 10 year survival of 84 and 62% respectively [3]. The data in adults in the 18–45 age range [more relevant to the adult IMD population] are even better with 1 year survival is 90% and 10 year survival 74%] in patients transplanted for all indications [3]. Quality of life following liver transplantation approaches that of the general population and remains superior to the preoperative status up to 20 years post LTx [4].

Risks of LTx are related to the surgical procedure [bleeding, vascular or bile duct injury] and the immunosuppression [infection, rejection, malignancy]. The type of graft impacts risks such as early allograft dysfunction and biliary complications, so use of a living donor graft or avoidance of expanded criteria donors for cadaveric grafts is preferred [5]. Living donor grafts are especially useful in IMDs as the procedure can be timed to interventions to optimize metabolic control. In some [but not all] IMDs, use of heterozygous living related donors may not be advisable [6]

There are additional early LTx risks of mortality and morbidity related to the IMD itself such as metabolic decompensation. However, patients with IMDs who do not undergo LTx are also at risk of metabolic decompensation and, when comparing survival of patients [children and adults] transplanted for MMA, PA, MSUD and UCD in Europe, data suggest that LTx offers a chance of significant disease modification or cure with a survival rate that is similar to [or better than] patients managed medically [7]. Mortality after transplantation is highest in the first 14 days after transplantation in this specific patient group, due to both metabolic decompensation and transplantation specific complications [7].

### LTx in target IMDs

The impacts of LTx vary amongst the target IMDs [sample studies shown in Table 1]. More complete lists are available for interested readers in recent systematic reviews [12, 14, 15, 17–20].

**Table 1:** Selected outcome studies of liver transplantation in inherited metabolic diseases

Disorder	Study	Total N **N** > **18 years**	1 year patient survival	5 year patient survival	Complication rate	Comment
UCD	Kim 2013 [8]	N: 23 *N* ≥ 18 years: 0	100%	100%	Rejection 35% Hepatic artery thrombosis 4% Bile tract damage 13% Retransplantation 5%	Study specifically looking only at pediatric patients
	Kido 2017 [9]	N: 42 *N* ≥ 18 years: 7	100%	100%	Not reported	All live donor LTx; 5 year survival of UCD patients not undergoing LTx 89.3%
	Yu 2015 [10]	278 *N* ≥ 18 years: 13	93%	89%	Graft loss in 21%	Data collected 1987–2010
GSD1a	Maheshwari 2012 [11]	95 *N* ≥ 18 years: Not reported; mean age of LTx group 17.7 years	88%	76%	Not reported	Data collected 1986–2007 Includes multiple types of GSD Survival of GSD patients better than matched control group of patients undergoing LTx for other disorders
	Beyzaei 2023 [12]	210 *N* ≥ 18 years: 21	Not reported	Not reported	GSD related complications post LTx 22.2%; Transplant related complications 27.7%	Metanalysis of 14 studies dating to 2003; includes multiple types of GSD
PA	Alexopoulos 2020 [13]	23 [PA] *N* ≥ 18 years: 0	89.5%	89.5%	5 year graft survival 84.6% No events of vascular injury	Graft and patient survival did not differ between PA and non-PA patients
	Zhou 2021 [14]	70 [PA] *N* ≥ 18 years: 1 [+1 16 years old]	Patient survival 95%; allograft survival 91% but median length of follow up not reported		Rejection 20% Hepatic artery thrombosis 8% Biliary complications 3%	Meta-analysis with studies dating to 2002
MMA/PA	Chakrapani 2023 [15]	70 [PA] 88 [MMA] *N* ≥ 18 years: 14 [+2 17–18 years old]	PA 81% survival at median 2.2 years follow up MMA 97.7% survival but follow up interval not reported		Hepatic artery thrombosis in 15% of PA; complication rates for MMA not systematically reporte9	Systematic review; MMA data combines isolated LTx with those who also had liver-kidney transplants
	Chu 2019 [16]	18 [MMA] 2 [PA] *N* ≥ 18 years: Not reported	94.4% [MMA] 100% [PA]	83.3% [MMA] 100% [PA]	Not reported	Survival was better for MMA patients who got LTx than for those who did not
MMA	Jiang 2021 [17]	109 [MMA] *N* ≥ 18 years: 3	99.9%	Not reported for entire cohort	Biliary complications 0.2%; vascular complications 7.7%; rejection 18.4%	Meta-analysis with studies dating to 1998

Selected outcome studies showing the impact of LTx on target IMDs. Single center studies in this table were chosen to prioritize data from the tacrolimus era of transplantation but some longer term studies include data which predates the modern era of transplantation which may result in an underestimate of the benefits of LTx; meta-analyses, systematic reviews and data from large transplant registries may contain data which predates this era

In symptomatic patients with UCD, LTx improves survival [21], dramatically reduces episodes of hyperammonemia [8, 22] and improves quality of life [23] relative to patients managed medically. Developmental outcomes are impacted by the age of transplantation [24] and this effect is most pronounced in patients with more severe disease and peak ammonia concentrations above 300 umol/L at presentation [9]. Survival rates (Table 1) for UCD patients after LTx are high [~90% at 5 years] which compares favorably with reported survival rates for patients with neonatal onset UCDs undergoing medical management [25]. Clinical practice guidelines describe LTx as “curative” for certain types of UCD and recommend that this intervention be considered for patients with severe phenotypes [26]. In adult patients with milder phenotypes, LTx is the only treatment which can address both the disease and its hepatic complications such as acute liver failure, hepatocellular carcinoma [HCC] and cirrhosis [27]. Women with UCD are at increased risk of metabolic decompensation during pregnancy and post partum [28] and increasing experience with pregnancy following LTx [29] means desire for pregnancy may be an indication to consider LTx.

The benefits of LTx on health outcomes GSD1a are also impressive with elimination of hypoglycemia [30], improvement in metabolic abnormalities [31], and quality of life [30]. LTx can also treat adenomas or HCC in GSD1a [32]. Patients may develop renal dysfunction after LTx [31] but the rate is although at similar rates to other non-renal pediatric disorders requiring LTx such as like biliary atresia [33] and may thus not always be GSD related. Biopsy evidence to understand the nature of the renal dysfunction [e.g. related to GSD1a or to toxicity of immunosuppression etc.] would be useful but is often not reported [31]. Patients with GSD1a undergoing LTx may have better outcomes than patients with other types of diseases who require LTx [11]. Consensus guidelines [34] suggest that LTx is should mainly be considered for GSD1a patients with adenomas but can also be considered as a means to improve quality of life.

The benefits of LTx on MMA and PA are more nuanced as LTx can modify the phenotype but is not curative. LTx is recommended for patients with frequent metabolic decompensation [35] as it reduces hospitalizations [16, 36]. Diet is liberalized [14] but not normalized in most patients with improvement in caregiver anxiety [15, 16] and quality of life [37]. Chu et al. [16] reported that the survival of the patients who underwent LTx was improved relative to those who did not undergo transplant [5 year survival 83.3% for LTx; 75% for medical management] although it is not clear how comparable the patients are in the two groups [16]. LTx in patients with early renal impairment related to MMA may improve renal function but combined liver-kidney transplant is required for patients with more advanced chronic kidney disease [38]. Not all IMD related complications respond in the same way to LTx – for example, cardiomyopathy improved in some PA patients post LTx [14, 15] but developed in others [39].

The impact of LTx on neurological complications of MMA and PA is complex. Some reports suggest non-significant trends to improvement in developmental outcomes post LTx [39, 40]. New neurological complications, which could be related to the IMD have been reported post LTx including Leigh syndrome [36] and metabolic stroke [39]. A number of reports document neurological findings consistent with calcineurin inhibitor [CNI] toxicity and early efforts to identify this and reduce or discontinue the CNI are indicated [7]. A literature review by Molema et al. [7] suggested that the frequency of CNI induced neurotoxicity in patients with MMA was similar to that reported for other patient groups undergoing LTx, with the exception of the incidence of posterior reversible encephalopathy syndrome which seems to be reported at a higher rate in MMA patients [7].

## Gene therapy medicinal products

### General information on GTMP

There are few published human trials available for GTMP on our target IMDs but informative data are available on GTMP in other conditions which may add to the information available for our target conditions.

### Safety risks with GTMP - focus on systemic adeno-associated virus [AAV] gene therapy and mRNA-lipid nanoparticle therapy

AAV mediated gene therapy has known limitations and risks but many uncertainties remain. A major limitation is that 40 to 80% of adults have pre-existing anti-AAV antibodies, for whom attempts of therapy in some diseases may be higher risk or even futile [41, 42]. Adaptive humoral immune responses and innate responses [e.g. complement activation] can occur, leading to loss of transduced cells or tissue damage from a cytotoxic T-cell response to the new gene product [43] although the impact of these antibodies on therapeutic efficacy of GT may vary in different diseases. The product monographs for currently approved systemically-delivered AAV gene therapies [onasemnogene abeparvovec, etranacogene dezaparvovec, valoctocogene roxaparvovec, and delandistrogene moxeparvovec] include warnings of the risk for serious liver injury. Hepatoxicity peaks several weeks to months after dosing and has been a cause of death [44, 45]. High vector dose and pre-existing liver disease are risk factors for hepatotoxicity [44, 46] which is a theoretical concern in patients with IMDs associated with liver disease such *as* GSD1a or UCD. Thrombotic microangiopathy and dorsal root ganglion injury are other immune-mediated injuries reported after AAV GTMP [47, 48]. Genotoxicity leading to tumor development has been reported in animal models receiving AAV gene therapy [49], particularly in mice with increased rates of HCC [50]. Cancers confirmed secondary to AAV-gene therapy have not yet been reported in humans but this risk needs to be closely evaluated in patients who are at higher baseline risk [e.g. GSD1a]. Cancers with non-AAV gene therapies have been reported in humans 5 or more years after administration [51] and others] which is why follow up to 15 years post administration is suggested by some regulatory agencies [52]. Some labels advise there may be a theoretical increased risk for HCC and recommend alpha fetoprotein marker and ultrasound monitoring in patients with additional risk factors [53].

There are many strategies being developed to mitigate safety risks such as various immunosuppression protocols and use of tissue specific promoters and other elements to drive higher expression and thereby allow lower vector dose [54]. While data on the safety of AAV-based GTMP are recent and therefore relatively limited, there is a large data set published by, Novartis in 2024 which describes over 4000 children who had received Zolgensma, a systemically delivered gene therapy using AAV9 [55]. This high number of treated patients with relatively low numbers of serious adverse events suggests the risk-benefit profile is balanced toward benefit in this severe disease. The risk-benefit balance may be more difficult to judge for IMDs that may be milder or have other effective therapies. In IMDs with the potential for pre-existing liver injury, such as urea cycle disorders or GSD1a, a cautious approach to surveillance for hepatotoxicity or hepatic tumors post AAV gene therapy may be warranted, especially in late-treated adults.

The safety profile of the mRNA-lipid nanoparticle (mRNA-LNP) treatment platform has been well established since billions of doses of the mRNA COVID-19 vaccines have been administered. Fewer than 1 in 1000 recipients experience a severe acute allergic reaction [56]. In the published data on mRNA-LNP therapy for propionic acidemia, only one grade 3 drug-emergent reaction [pancreatitis] was observed in the 16 participants; many others experienced milder events, including infusion-related reactions [57].

### GTMP in target IMDs

Table 2 outlines select clinical trials for GTMP in our target IMDs. There are few available full publications [although some data are available in abstract form] so data are currently limited on efficacy, safety, and outcomes. Publications lag behind data availability so updates are also available through sources of information such as ClinicalTrials.gov and the Gene Therapy Clinical Trials database https://www.genetherapynet.com/clinical-trials.html.

**Table 2:** Selected gene therapy medicinal products trials in target inherited metabolic diseases

Disorder	Study name and Clinical Trials Number	Phase	Description	Duration of follow up	Published data in humans
OTCD	Ultragenyx DTX301 NCT 05345171 [58];	3	AAV gene replacement in patients with late onset OTCD ≥ 12 years of age	64 weeks	Phase1/2 published in abstract form [59]
	iEcure ECUR-506 NCT06255782 [60]	1/2/3	AAV gene editing in male infants with neonatal onset OTCD	24 weeks	Data on first patient published in abstract form [61]
	Arcturus NCT06488313 [62]	1/2	mRNA dose ranging trial in symptomatic OTCD ≥ 12 years of age	85 days	Phase 2 published in abstract form [63]
GSD1a	Ultragenyx DTX401 NCT05139316 [64]	3	AAV gene replacement placebo controlled cross over trial >8 years of age	144 weeks	Phase 1/2 published [65]
	BEAM 301 NCT06735755 [66]	1/2	Base editing in LNP in adults	24 months	No
	Moderna mRNA-3745 NCT05095727 [67]	1/2	LNP mRNA in multiple age groups	3.5 years	Abstract form [68]
PA	Moderna mRNA-3927 NCT04159103 [69]	1/2	LNP mRNA in multiple age groups	150 weeks	Phase 1/2 published [57]
MMA	Moderna mRNA-3705 NCT04899310 [70]	1/2	LNP mRNA in patients > 11 kg	144 weeks	Abstract form [71]

Select GTMP trials in humans for target IMDs. Available publications were determined through PubMed search and review of selected conference proceedings so listing may be incomplete related to delays before presented or published work becomes available on PubMed

#### UCD – focus on ornithine transcarbamylase deficiency [OTCD]

GTMP studies are most advanced for OTCD with three GTMP at later stages of development:

Ultragenyx DTX301, is an AAV gene replacement therapy now in Phase 3 trials [59]. An abstract from the phase 1/2 trial documents complete response [discontinuation of scavengers and protein restriction] in 4 of 11 participants [although one of these was reclassified as a partial responder at week 182] with partial response [>50% reduction in medications and dietary protein restriction relative to baseline] in 3 subjects [59]. Six patients required corticosteroids for transaminitis. The AAV vector utilized in this therapy is primarily non-integrating so questions remain regarding the duration of the therapeutic benefits that patients may experience following treatment, concerns which are consistent with pre-clinical studies of AAV encoded OTC activity showing that administration in newborn mice did not provide durable control of hyperammonemia through adulthood [72]. A phase 3 trial of DTX301 is in progress with positive findings in a press release [73] but not yet published.

Phase 2 human data are available in abstract form for ARCT-810, developed by Arcturus Therapeutics, mRNA-based therapy for patients with OTCD given as lifelong biweekly infusions showing reduction in glutamine levels [63]. No published human data are yet available to evaluate iEcure’s ECUR-506 gene editing therapy [60], which employs AAV as a delivery vehicle. This treatment is being targeted to male infants diagnosed with severe neonatal onset OTCD, but it may not be suitable for older children or adults due to the associated risks of hepatotoxicity. Some positive data on the first infant treated with this product have been published in abstract form [61]. Another trial of an AAV-based therapy for OTCD sponsored by University College, London is targeted to children and evaluating this therapy as a potential bridge to transplant [74].

#### GSD1a

Utragenyx DTX401 is an AAV8 gene therapy for GSD1a now in phase 3 trials. Preliminary results from phase 1/2 dose escalation study have been published [65] suggesting DTX401 was relatively safe with no dose limiting toxicity. However, prednisolone doses (40–60 mg/day) were required for a mean of 90 days in 11 of 12 patients which is a concern in this patient population with increased baseline risks of osteoporosis and insulin resistance. Long term follow-up data for this product should include the risk of steroid-related related sequalae such as fractures and development of diabetes mellitus. The data on efficacy are challenging to interpret given multiple protocol changes discussed elsewhere. However, daily cornstarch intake did decrease and most participants noted improvements in quality of life. Data from 46 subjects in the phase 3 trial placebo controlled cross over trial of DTX401 available in abstract form [75] confirm reductions in cornstarch dose and success of the protocol for prophylactic steroid management in preventing liver enzyme elevation.

BEAM-301 [Beam Therapeutics], a gene correction approach using lipid nanoparticles [66], is in phase 1/2 trials, with published preclinical data but awaiting published data in humans. The abstract from a poster presentation on a phase 1/2 study of Moderna’s lipid nanoparticle [LNP]-encapsulated mRNA-based therapeutic agent [mRNA-3745] suggested improved time to hypoglycemia [68].

For the one product with published data [DTX401], the results, while promising, do not suggest that the efficacy of this therapy will approach that of LTx. The main indications for LTx in adults with GSD1a are liver adenomas or HCC where GTMP are not expected to be treatment alternatives. However, it is possible that GTMP may impact the risk of development of these complications although long term data will be required to confirm this. Of interest, one gene therapy study using a lentiviral vector given to neonatal mice showed a marked reduction in the risk of development of hepatic tumours in a GSD1 mouse model [76] but no human trials of this agent appear to be in progress.

#### MMA/PA

The GTMP in clinical trials for these organic acidurias are RNA based LNP which require repeated administration. Interim results from the trial of mRNA-3927 in PA suggest a reduction in episodes of decompensation requiring emergency care [57]. Safety concerns such as infusion related relations were manageable although 1 patient had grade 3 pancreatitis [57]. At a median of 83.9 weeks of follow-up, 18 patients treated with mRNA-3705 for MMA also had reductions in the risk of metabolic decompensation and hospitalization with no dose limiting toxicities [71] but 3 of the 18 patients discontinued the extension trial for reasons not specified in the abstract.

### How do published data on LTx compare to those of GTMP in terms of data quality?

#### Evaluated outcomes

Trials of new therapeutics evaluate outcomes relative to medical management, but this might not be the appropriate comparator in disorders where LTx can be curative [UCD] or have marked effects on phenotype [GSD1a, MMA]. As it is not feasible to design a trial where patients are randomized to a new therapeutic such as a GTMP or LTx, health technology assessment must separately evaluate the benefits and risks of the individual therapies. GTMP are in earlier phases of development so trials are focused on safety and surrogate markers. As these trials advance, information on hard endpoints [as are available for LTx – Table 3] in both adults and children will be needed to allow comparison. If data are only available in certain age groups, some patients may not be able to access these therapies through publicly funded health plans even though they may benefit. In many rare diseases, new drugs are often marketed lacking data on hard clinical endpoints [77]. Data on hard outcomes are easier to achieve for the most severe forms of acute disorders [such as the intoxication type IMDs] than for milder phenotypes or chronic disorders like GSD1a. Managed entry agreements can be useful in situations where meaningful outcome data requires very long term follow up which is not feasible in a clinical trial setting.

**Table 3:** Published outcome data for liver transplantation in target inherited metabolic diseases

Outcome	UCD	GSD1a	MMA/PA
Survival	+	+	+
Hospitalizations	+	+	+
Neurologic outcomes	+	+	+
Growth	+	+	+
Dietary relaxation	+	+	+
Quality of life	+	+	+
Biochemical abnormalities	+	+	+

Types of outcome data available for LTx in target IMDs to illustrate what data will need to be available in future for GTMP to allow health policy makers to compare these different treatment modalities

#### Duration of follow up

LTx is a long-established treatment modality and data are available on hundreds of UCD [10] and GSD [12] patients going back more than 4 decades. Duration of follow up for other conditions like MMA/PA is shorter, with one 2021 meta-analysis documenting that only 30% of the published patients had data extending at least 3 years post LTx and only 12% had data of 5 years or more [17]. However, evaluating publications with long term follow up may actually complicate interpretation of the benefits of LTx as the year of transplantation impacts outcomes. Ashwat et al. [78] documented improved 90 day, 1 and 5-year survival when evaluating the transplant related outcomes from 2002 to 2018 despite worsening donor and recipient risk profiles. These improvements relate to improvements in surgical techniques, general medical care, organ preservation, and immunosuppression. Some of the larger studies [79, 80] include patients who were transplanted long before these improvements. So, although there are much more long-term data available on LTx than for GTMP, not all of these long-term data reflect advances in transplantation-related care which could result in underestimation of the benefits of LTx in IMDs.

Although gene therapy for UCDs was first trialed in UCDs in the late 1990s, the death of one of the trial participants paused development of GTMPs for UCD for some years [20]. Therefore, the GTMP currently in development for our target IMDs are recent developments with published follow-up measured in weeks or months (Table 2). The short duration of follow up is particularly an issue with respect to malignancy risk as discussed. Longer term data will be forthcoming for these trials but it is likely that some of these GTMP in development will be granted market authorization before this information is available.

#### Methodological considerations

It is difficult to study rare diseases. We give some examples of methodological concerns not to be critical, but rather to help clinicians understand the limitations of the data. There are a number of common concerns with studies on LTx in our target IMDs (Table 1). In addition to the issue of transplant era [discussed above], some studies combine data from LTx with that of combined liver kidney transplant [e.g. 34 and others] or do not distinguish by donor type [10]. Some studies combine survival data for adults and children [10] or for multiple different disorders [81]. There are quite a few review papers and yet a relatively small number of cases worldwide so it is uncertain where cases in publications may overlap.

There are also methodologic issues in the few published studies of GTMP in our target IMDs, some of which arise from learning based on response of early patients to these novel therapies. For example, multiple protocol changes were needed in the Phase 1/2 trial of DTX401 for GSD1a [65]. There were changes in definition of hypoglycemia, the pretest meal composition, pre-test cornstarch dose, and stopping criteria which make the information on the increase in time to hypoglycemia [one of the trial endpoints] hard to interpret. It is difficult to assess methodologic issues in studies of GTMP which are currently only in abstract form [59, 65, 71].

### Health policy considerations differ between children and adults

Most of the data available on LTx (Table 1) are for children. The age indications (Table 2) for the clinical trials of the various GTMP differ so we can anticipate age gaps in future data. Focusing research on children is understandable, given that data suggest early intervention results in the best neurological outcomes for the intoxication type IMDs [82] so if patients are treated as children, adult treatment will not needed. However, real world challenges mean that many adult patients with phenotypes severe enough for LTx or GTMP are not treated as children and may benefit from such therapy. Data suggest that already more than 50% of patients with IMDs are over the age of 18 [83], a number which is increasing over time.

Another challenge is access to pediatric LTx which is more limited than for adults [84] and recent data suggest the annual number of pediatric LTx being performed in Europe is decreasing [85]. Also, LTx has been used more extensively for some IMDs [like UCD] than for others [like organic acidurias]. McKiernan et al. [1] tracked transplants in the U.S. over 3 decades and found that only 20 transplants in total for organic acidemias were done prior to 2007, so many such patients reached adulthood without this therapy.

Finally, safety and efficacy are very likely to differ for children and adults given their different comorbidity profiles. For example, the degree of hepatic glycogen storage [related in part to patient age] may alter the impact of GTMP on the risk of hepatic adenoma/carcinoma in GSD1a. Age specific data are needed for decision making but are unlikely to be available at the time of market authorization which may lead to reimbursement criteria which restrict the use of GTMP to a narrow age range, creating inequities for patients of other ages who may potentially benefit.

### Health policy impacts of access and opportunity costs

Limited availability of donor organs is the Achilles heel of solid organ transplantation. Increase in the use of live donors has expanded the available organ pool but uptake is slow in some countries and gaps still remain between the number of patients awaiting transplant and available organs [86]. Live donor transplantation is less common in adults [87] and access to LTx adults with IMDs may be even worse because of lack of familiarity of transplant teams with the conditions and organ allocation policies that prioritize synthetic liver dysfunction [2]. Alternative treatments, such as GTMP, are not the only way to reduce the gap between available organs and recipients who need them. Policies which increase rates of organ donation or health measures which reduce the risk of liver disease are also needed. In theory, GTMP, which are not impacted by organ availability, should be more accessible than solid organ transplantation although accessibility may be impacted the type of GTMP [e.g. related to pre-existing antibodies to the vector etc.]. However, worldwide access limitations for GTMP are even more severe than for LTx due to both the high price of GTMP and lack of infrastructure for the administration of these complex therapies [88]. The fact that GTMP are almost exclusively limited to high income countries [88] is an ethical concern leading one Nobel prize winning scientist to state“… the people who could benefit most won’t be able to access or afford them” [89].

The high opportunity costs of GTMP also pose ethical challenges for those countries that do reimburse them. To our knowledge, there are not studies that directly compare the cost effectiveness of GTMP to that of LTx in our target diseases but there are studies that compare each form of treatment to conventional medical management. Bretos-Azcona et al. [90] modelled mRNA based therapies for MMA and PA and concluded these would add 5.7 and 1.3 quality adjusted life years [QALYs] respectively when added to conventional therapy. These added QALYs came at substantial additional cost [-£337k/QALY in MMA and £2.53 M/QALY in PA] leading the authors to conclude that these therapies would need to be priced below £100k per year to meet even the more liberal willingness to pay thresholds used for rare diseases [£300k/QALY]. By contrast, an earlier study by Li et al. [91] suggested that LTx would result in a gain of 7.9 QALYs for patients with PA/MMA and lead to a lifetime net cost *savings* of $USD582k. This number reflects cost savings of LT in the need for nutritional support as well as indirect costs such as need for special education and loss of productivity which more than offset the increased direct medical costs of LT. This study did not include the costs of newer medications like carglumic acid for PA so the cost savings associated with LTx would be even greater in patients who require this expensive therapy.

We could not find similar data which provides specific estimates of QALYs gained and cost/QALY for LTx or GTMP in UCD or GSD1a. However, the cost of LTx is highest in the first year and declines thereafter. Hospitalization costs for the LTx procedure vary by country but the average cost of the inpatient component of the procedure in Canada ranges from $CAD75k to $CAD170k [92]. This estimate does not include outpatient care costs. The costs for GTMP in development for the diseases we discuss here are not yet known. However, available forms of GTMP for other diseases are associated with much higher costs [>$100k/year for ongoing therapies and $1-5 M for available single use therapies] so these therapies may never challenge LTx in terms of cost effectiveness.

The costs for some medications used in IMDS [such ammonia scavengers] are very high. Any calculation of cost effectiveness of LTx and GTMP for conditions like UCD is distorted by the high costs of these medications although there is not consensus on the best way to adjust for these distortions. This situation is described by economists as “theory of the second best” [93] where the price of a therapy no longer reflects its societal value but simply reflects the distorted market. In conditions where the impact of LTx is large [MMA, UCD], LTx may actually be a more appropriate comparator then medical management. Such an approach would negatively impact the cost-effectiveness profile GTMP [as LTx is cheaper than lifelong use of expensive medications] but would more accurately reflect the decisions facing health policy makers. This approach would be difficult though for patients and clinicians given that a GTMP infusion has much less short term risk than LTx.

There are many proposed policy options to improve the cost-effectiveness of novel therapies such as GTMP [94, 95] but at the current time, LTx may be more cost effective option using traditional metrics relative to GTMP as long as such prices remain uncontrolled. As the GTMP for our target IMDs seek market authorization and pricing becomes known, LTx, rather than medical management, should be considered as the comparator at the time of health technology assessment to allow comparison of the relative cost-effectiveness for these different modalities.

Knowledge about relative cost-effectiveness, while important, is only one of several policy considerations and the literature suggests that traditional metrics such as cost per QALY are difficult for policy makers to apply in rare diseases [96] where the quality of the evidence to available to assess drug effectiveness may be low and the prices are determined by non-market forces such as willingness to pay [97].

Despite these challenges, some standardization must occur about which treatment modalities are reimbursed by strained publicly funded health care systems. The decisions facing health policy makers differ from those facing patients. The choice of GTMP or LTx for patients may come down to known risks versus risk of the unknown. It is clear that patients and families respond differently in evidence-poor environments. Gerstein et al. [98] collected qualitative data from parents choosing between conventional medical therapy and LTx for UCD. There were common themes [disease severity, burden on family, access to care, etc.] but within these common themes, different families made different decisions. Tolerance for risk is lower when parents of younger children are asked relative to parents of older children or when adults are asked for themselves [99].

Health policy makers, however, must balance patient preferences with competing priorities, ensuring that funded treatments are sustainable, and provide value for investment as well as equity in the treatment of different patient groups. Patient choice is one consideration but it is not routine practice to allow patient choice to be the sole factor influencing which treatments are funded. For example, it is routine in oncology for therapies to be funded in order [“first line, second line etc.”] rather than at the discretion of the patient. While input is obtained from stakeholders such as patients, families, health care providers, and manufacturers on decision making, it is not routine to seek input of the public even though the public are significant stakeholders in such discussions. Decisions made for IMD patients could directly impact resources [funding, organs] available for patients with common diseases. Public attitudes can be assessed using tools such as surveys [100], discrete choice experiments [101], or using public values to inform criteria used in multi-criteria decision analysis, which is one of the most common tools used by policy makers for orphan drugs [102]. Research has shown that the public are more concerned about issues such as cost of care and management of risks than scientists [101, 103] resulting in an urgent need for studies which evaluate the attitudes of the public on highly relevant questions such as:

- Should resources [such as public investments in research, hospital infrastructure, funding etc.] for GTMP be targeted only to those conditions where other curative options [such as LTx] are not available?
- How should patients with alternative treatment options such as GTMP be weighted relative to those with no other therapeutic alternatives in terms of priority for the limited pool of liver transplants?
- What parameters should be considered when choosing between different treatment options for a rare disease are available with similar efficacy on disease manifestations but that differ significantly in risk and/or cost?
- How should the policy makers evaluate treatments with high risk and/or opportunity costs in chronic conditions where therapeutic interventions may not increase quantity of life but could improve quality of life such as GSD1a?

## Conclusions

Promising GTMP are in development for our target IMDs but data on their full impacts on patient outcomes [both positive and negative] are not yet available. However, even at these earlier stages of development, we can make some inferences when comparing GTMP and LTx for our target conditions:

- Based on the limited existing data, none of the GTMP discussed here appear to be curative but do provide disease modification to a greater [MMA, UCD] or lesser [GSD1a] extent. LTx provides marked benefits [MMA, GSD1a] or even cure [UCD] with similar [or in some situations better] survival rates to patients who remain on medical management but data comparing survival of LTx with GTMP are not available. The established benefits of LTx in our target IMDs make this modality a more appropriate comparator for health technology assessment than nutritional management when considering the impact of future GTMP.
- There are data quality issues with published information on both LTx and GTMP. However, data are available on hard outcomes [hospitalization, cognitive status, survival] as well as quality of life for LTx and such data in both adults and children will be required for health policy decision makers considering reimbursement of GTMP.
- Organ availability restricts access to LTx but access issues for GTMP are even greater at the present time, albeit potentially remediable through actions to control price.
- Data for both LTx and GTMP are more limited in adults than in children and it is necessary to prioritize research in the adult age group.

The rapid development of innovative GTMP is exciting but also raises significant challenges for health policy makers. These challenges are magnified in adults which means that LTx in the era of GTMP is not obsolete but, for some patients, may be needed more than ever.

## Data Availability

Not applicable.

## Abbreviations

AAV: Adeno-associated virus
CNI: Calcineurin inhibitors
GTMP: Gene therapy medicinal products
GSD: Glycogen storage disease
HCC: Hepatocellular carcinoma
IMDs: Inherited metabolic diseases
LNP: Lipid nanoparticle[s]
LTx: Liver transplantation
MMA: Methylmalonic acidemia
OTCD: Ornithine transcarbamylase deficiency
PA: Propionic acidemia
QALY: Quality-adjusted life year
UCD: Urea cycle defect
